# Analysis of static parameters in retrospective studies: limitations and interpretation

**DOI:** 10.1186/s13054-023-04691-4

**Published:** 2023-10-23

**Authors:** Yanfei Shen

**Affiliations:** Department of Intensive Care, Zhejiang Hospital, 1229 Gudun Road, Hangzhou, 310013 Zhejiang China

To the Editor,

We read with great interest the recent study [1] by Dr. McGuigan et al. A total of 32,349 patients after cardiac arrest were included, and both the lowest and highest mean arterial pressure (MAP) and systolic blood pressure (SBP) were analyzed. According to the results from restricted cubic splines analysis, they reported nonlinear associations between MAP and mortality: the lowest MAP within the 60–63 mmHg range had the lowest mortality and the highest MAP within the 95–104 mmHg range had the lowest mortality. A similar pattern was also found for SBP.

The strength of the current study is the large sample size and statistically stable non-linear associations using restricted cubic splines analysis. This study is well designed. However, several limitations should be noted. First, unlike laboratory indexes, physiological parameters such as MAP, heart rate, and respiratory rate are recorded continuously in critical care, and some extreme values are very susceptible to interference by various factors. For instance, some common procedures in the critical care unit, such as sputum aspiration or various catheter catheterizations, often lead to transit but rapid increases in blood pressure. Meanwhile, other situations, such as artery catheter distortion and excessive use of sedatives, can also lead to transit but low blood pressure records. Therefore, static extreme MAP values (both lowest and highest) are not enough to accurately reflect the actual “long-term” blood pressure in critically ill patients. This may be the main difference between randomized trials [2] and retrospective studies, as blood pressure is often not under intensive control, and the variability is often large in retrospective design, which may also be the reason for these different findings [1, 2]. However, for critically ill patients, blood pressure during most of the time, rather than some extreme static MAP, may be more relevant to prognosis.

Second, in the current study, the lowest and highest MAP were analyzed using restricted cubic splines analysis, and both the lowest MAP in the range of 60–63 mmHg and the highest MAP in the range of 95–104 mmHg had the lowest associated mortality. The statistical results were stable. However, the interpretation is a little confusing. For instance, according to the result of the lowest MAP, the lowest MAP higher than 63 mmHg was associated with high mortality. However, according to the result of the highest MAP, the highest MAP lower than 95 mmHg was also associated with high mortality. Then for a patient with MAP ranging from 75 to 80 mmHg, what should we do [3]? Of course, we agree with the authors’ opinion that due to the retrospective nature, the causal relationship cannot be inferred. We suggest more trials are needed to explore the “real” threshold of MAP in patients after cardiac arrest [4].

Finally, this study contributed greatly to the threshold of MAP in patients after cardiac arrest, and this work is very much appreciated!

## Data Availability

Not applicable.
